# Conjugative IncFI plasmids carrying CTX-M-15 among *Escherichia coli *ESBL producing isolates at a University hospital in Germany

**DOI:** 10.1186/1471-2334-9-97

**Published:** 2009-06-17

**Authors:** Stephen E Mshana, Can Imirzalioglu, Hamid Hossain, Torsten Hain, Eugen Domann, Trinad Chakraborty

**Affiliations:** 1 Institute of Medical Microbiology, Justus-Liebig University, Giessen, Germany; 2 Department of Microbiology, Weill Bugando University College of Health Sciences Mwanza, Tanzania

## Abstract

**Background:**

Multi-drug-resistant, extended-spectrum β-lactamase (ESBL)-producing Enterobacteriaceae, constitute an emerging public-health concern. Little data on the molecular epidemiology of ESBL producing *Escherichia coli *is available in Germany. Here we describe the prevalence and molecular epidemiology of ESBL producing-*Escherichia coli *isolates at a German University hospital.

**Methods:**

We analysed 63 non-duplicate clinical ESBL isolates obtained over an 8-month period using PCR and sequence-based ESBL allele typing, plasmid replicon typing, phylogenetic group typing. Pulsed-field gel electrophoresis (PFGE) based genotyping and plasmid profiling was performed, as well as confirmatory DNA-based hybridization assays.

**Results:**

Examination of the 63 *Escherichia coli *isolates revealed an almost equal distribution among the *E. coli *phylogenetic groups A, B1, B2 and D. High prevalence (36/63) of the CTX-M-15 gene was observed and an analysis of PFGE-based patterns revealed the presence of this CTX-M allele in multiple clones. Resistance to cefotaxime was a transferable trait and a commonly occurring 145.5 kb conjugative IncFI plasmid was detected in 65% of *E. coli *carrying the CTX-M-15 allele. The rate of transferable antibiotic resistances for GM, SXT, TET, GM-SXT-TET, SXT-TET and GM-TET was 33%, 61%, 61%, 27%, 44% and 11%, respectively. The remaining strains did not have a common IncFI plasmid but harboured transferable IncFI plasmids with sizes that ranged from 97 to 242.5 kb.

**Conclusion:**

Our data demonstrate the presence of IncFI plasmids within the prevailing *E. coli *population in a hospital setting and suggest that the dissemination of CTX-M-15 allele is associated to lateral transfer of these well-adapted, conjugative IncFI plasmids among various *E. coli *genotypes.

## Background

Emergence of resistance to β-lactam antibiotics was described even before the first β-lactam penicillin was developed. The first β-lactamase was identified in *E. coli *prior to the use of penicillin in medical practice [1]. In 1983, a *Klebsiella ozaenae *isolate from Germany was found to secrete a SHV-2 β-lactamase which efficiently hydrolyzed cefotaxime and to a lesser extent ceftazidime [2]. Many ESBL-producing enterobacterial isolates express enzyme variants that are derived from TEM-1 and SHV-1 by mutations. Recently, different types of ESBL such as CTX-M have been detected. These enzymes hydrolyze cefepime with high efficiency and cefotaxime more efficiently than ceftazidime [3]. In clinical strains, CTX-M-encoding genes have commonly been located on plasmids which vary in size from 7 – 200 kb [3-5]. A number of studies have established that most of these plasmids have either replicons that belong to the incompatibility group (Inc) FII, or multireplicons of Inc FII associated with Inc FIA and FIB [4-8]. The presence of an Inc FI plasmid replicon harbouring CTX-M-15 was reported in a single isolate from Turkey [8]. Associations with plasmids of other Inc groups such as Inc I1 and Inc N have also been reported [7]. Many of these plasmids are conjugative and have transfer frequencies ranging from 10^-2^–10^-7^. Additionally, they also encode multiple resistance genes for different antibiotics as described for various Inc FII plasmids isolated in UK and Canada [9]. To date, more than 60 different CTX-M ESBLs belonging to 5 evolutionary groups have been described. In most clinical isolates CTX-M-15 is the most frequent CTX-M type, and has been reported in Enterobacteriaceae isolates from Poland, Canada, France, UK, Russia, Cameroon, India, Bulgaria and Japan [3-5]. In Germany isolates harbouring CTX-M-1, -3, -9 have previously been described [3].

In the present study, we prospectively examined *E. coli *ESBL-producing isolates at a German University Hospital with respect to their phylogenetic type, PFGE pattern, plasmid incompatibility groups and their antibiotic susceptibility profiles in order to shed light into the epidemiology of these isolates in our clinical setting. We report here on the emergence of a cluster of CTX-M-15 producers in Germany exclusively associated with a large conjugative IncFI replicon-type plasmid.

## Methods

### Isolates and susceptibility testing

A total of 63 non-duplicate ESBL-positive *E. coli *were isolated from clinical specimens obtained from different wards of the local university hospital. These isolates were obtained from urine, wound swabs, blood, sputum and aspirates. Polymicrobial infections with Enterobacteriaceae harbouring the same ESBL-type were detected in three cases of urinary tract infections (UTI): the first two cases involved *E. coli *and *Enterobacter cloacae *and *E. coli *and *Enterobacter gergoviae *in urine samples, while the third case involved *K. pneumoniae *in the urine sample and *E. coli *in blood culture of the same patient. This study was approved by the institutional review board of the University of Giessen and Marburg University Hospital and was deemed exempt from informed consent.

The specimens were collected over a period of 8 months from August 2006 to April 2007. Pure cultures of clinical isolates were identified based on biochemical test systems (API 20E, BioMerieux, France) following the instructions of the vendor. Antibiotic susceptibility was determined using the disk diffusion method on Mueller-Hinton agar (Oxoid, Basingstoke, England) as recommended by the Clinical and Laboratory Standard Institute (CLSI) [10]. Susceptibility was tested against ampicillin (10 μg), amoxycillin/clavunate (20/10 μg), ampicillin/sulbactam (10/10 μg), tetracycline (30 μg), gentamicin (10 μg), tobramycin (10 μg), SXT (1.25/23.75 μg), ciprofloxacin (5 μg), moxifloxacin (5 μg), cefpodoxim (10 μg), ceftazidime (30 μg), cefepime (30 μg), imipenem (10 μg) and meropenem (10 μg) (BD BBL, Franklin Lakes, USA). *Escherichia coli *ATCC 25922 and *Klebsiella pneumoniae *ATCC 700603 were used as reference strains.

All isolates resistant to multiple cephalosporins were confirmed for ESBL production using the double disk synergy method (disk approximation method) [10]. The MIC for cefepime and tigecycline in all isolates producing CTX-M alleles was determined using E-tests ranging from 0.016 to 256 μg/ml (AB Biodisk, Sweden) according to the manufacturer's instructions and the recommendations of the CLSI. Quality of media, antibiotic disks and E-test strips were controlled with the *E. coli *ATCC 25922 isolate. Isolates with a MIC of ≥ 8 μg/ml for cefepime and a MIC of ≥ 2 μg/ml for tigecycline were considered resistant according to the CLSI [10,11].

### Amplification of ESBL genes and the ISEcp1 element

The presences of genes encoding ESBL (CTX-M, TEM and SHV) were investigated using specific primers and methods described previously [12,13]. Using the published sequence of a 92 kb plasmid carrying CTX-M-15 GenBank accession NO AY044436 (9), primers *tnp*A/IS*Ecp*1 F (5'-GCAGGTGATCACAACC-3'), *tnp*A/IS*Ecp*1 R (5'-GCGCATACAGCGGCACACTTCCTAAC-3') and CTX-*tnp*A F (5'-CATGCTCACGGCGGG-3'), CTX-*tnp*A R (5'-GCTAGGTGATCACAACC-3') were designed to amplify the IS*Ecp*1 (1881 bp) and CTX-M-15 gene plus IS*Ecp*1 elements (3181 bp) in CTX-M-15-carrying *E. coli *and transconjugants [8]. For amplification, 5 μl of template DNA (50 ng/μl) was added to a 45 μl mixture containing 200 μM of dNTP mixtures (Roche, Switzerland) 0.4 μM of each primer, 2.5 U Taq polymerase (Invitrogen, Germany) and appropriate buffer (0.2 μM MgCl_2_, 2.5 μM KCL, 0.5 μl 10% Tween 20, 1 μl of Gelatin and 3.8 μl of pure water). The reaction was performed in a Gene Amp PCR system 9700 thermo cycler (Applied Biosystems, USA) under the following conditions: initial denaturation at 94°C for 5 minutes followed by 35 amplification cycles comprising of 30 seconds denaturation at 94°C, 30 seconds annealing at 62°C, 60 seconds extension at 72°C, followed by a final extension step at 72°C for 7 minutes. All PCR products were sequenced and a previously characterized *E. coli *J53-pMG 267 harboring bla_CTX-M _genes associated with IS*Ecp*1 was included as a positive control.

### Pulsed-field Gel Electrophoresis (PFGE) and phylogenetic group typing

PFGE was performed according to the Pulse Net protocol of the Centers for Disease Control and Prevention, Atlanta, USA http://www.cdc.gov/pulsenet/protocols.htm. The agarose-embedded DNA was digested with the restriction endonuclease *Xba*I (New England Biolabs, USA) at 37°C for 16 hrs. Electrophoresis was conducted using a CHEF Drive II (Bio-Rad, UK); conditions were 6 V, with 2.2 s–54 s pulses for 20 hrs. Strain differentiation by PFGE analysis was achieved by comparison of band patterns using Gelcompar II (Applied Maths, Belgium). Patterns were normalized using the molecular weight marker (PFGE Lambda Marker, Fermentas, Germany). Dendograms were generated to visualize relationships among the isolates. The similarity coefficient (SAB) of sample pairs was calculated based on band positions by using DICE metric [14,15]. A similarity coefficient SAB of 0.80 was set as a threshold for defining clusters of genetically similar isolates.

*Escherichia coli *phylogenetic grouping was achieved using triplex PCR for *chu*A, *yja*A and *tsp*E4C2 genes as described previously. Amplification of these genes was performed with the following primers *chu*A.1 (5'-GACGAACCAACGGTCAGGAT-3') and *chu*A.2 (5'-TGCCGCCAGTACCAAAGACA-3'), *yja*A.1 (5'-TGAAGTGTCAGGAGACGCTG-3') and *yja*A.2 (5'-ATGGAGAATGCGTTCCTCAAC-3'), and *tsp*E4C2.1 (5'-GAGTAATGTCGGGGCATTCA-3') and *tsp*E4C2.2 (5'-CGCGCCAACAAAGTATTACG-3') [5,16].

### Plasmid analysis, DNA hybridization and PCR-based replicon typing (PBRT)

Plasmids from transconjugants and clinical isolates were detected using PFGE as described previously [4,5,17]. Genomic DNA was prepared as described above. A single block was incubated at 55°C for 45 minutes with 1 unit of S1 nuclease (Invitrogen, Germany) in 200 μl of 50 mM NaCl, 30 mM sodium acetate and 5 mM ZnSO_4_. Electrophoresis was done under the following conditions 6 V, 5 s–50 s for 20 h. DNA fragments were transferred to the polyvinyl-based membrane using overnight capillary transfer followed by hybridization with digoxygenin (DIG)-labeled CTX-M-15 FIA, and FIB amplicon probes prepared according to the manufacturers instruction (DIG High Prime DNA labelling and Detection Starter Kit II, Roche, Germany) [5].

PCR-based replicon typing was carried out as described by Carattoli et al [5,7]. DNA was extracted using a DNeasy tissue extraction kit (Qiagen, Germany) and 5 μl of template DNA (50 ng/μl) was used in simplex PCR to detect FIA, FIB, FII, I1 and N groups. PCR conditions were: initial denaturation at 94°C for 5 minutes followed by 30 amplification cycles comprising of 30 seconds denaturation at 94°C, 30 seconds annealing at 58°C, 60 seconds extension at 72°C, followed by a final extension step at 72°C for 5 minutes. *Escherichia coli *strains ATCC 25922 and R 100 were used as negative and positive controls, respectively.

### Sequencing

PCR products of all ESBL genes detected as well as 6 randomly selected FIA- and FIB-amplicons were sequenced. PCR products were purified using Invitek purification kit (Invitek, Germany) following the manufacturer's instructions. Sequence reactions were done using the corresponding primers used for amplification, and sequencing was performed using the automated sequencer ABI Prism 3100 (Applied Biosystems, USA). DNA sequence was analysed using Lasergene software (DNASTAR, USA) followed by homology searches using the NCBI BLAST algorithm (Altschul SF et al. 1997).

### Transfer of antibiotic-resistance genes

Conjugation experiments were performed using *E. coli *CC118 (Rif^r^, Str^r^, Lac-, plasmid-free) as a recipient strain [18] and 18 randomly selected clinical isolates of *E. coli*, representing different PFGE-based clusters as donor strains. Strains were mixed at the ratio of 1:2 (donor/recipient) on LB agar followed by overnight incubation at 37°C. Transconjugants were selected by suspending the growth in 1 ml of PBS and 0.1 ml portions of 10^-1 ^to 10^-4 ^dilutions were plated on LB agar containing 300 μg/ml rifampicin and 30 μg/ml cefotaxime. The transconjugants were tested for ESBL production using disk approximation method followed by PCR amplification of ESBL genes and replicon typing.

## Results

### Isolates and types of ESBL genes

A total of 63 non-duplicate clinically relevant isolates of *E. coli *were confirmed as ESBL-producing strains using the double disk assay. Thirty five of these isolates were recovered from urine (55.5%), 6 from blood culture (9.5%), 5 from sputum (7.9%) and 17 were from swabs (wound, eye, cervix, 27%). Twenty-nine of the isolates originated from general medical wards (46%) and 7 were from intensive care units (11.1%). Among the 63 phenotypically confirmed ESBLs, 61 (96.8%) were positive in PCR amplification using primers specific for the detection of TEM and CTX-M genes. CTX-M occurred at the highest frequency and was found in 49 (77.7%) isolates. CTX-M-15 was the most common allele detected and was found in 36 (57.1%) of all *E. coli *tested. Other detected ESBL alleles were CTX-M-3 (4.7%), CTX-M-1 (11.1%), CTX-M-28 (3.1%), Tem-144 (7.9%), Tem-126 (3.1%), Tem-105 (3.1%), Tem-150 (1.6%) and Tem-143 (3.1%), '[see Additional file 1]'. CTX-M-15 was detected in all cases of polymicrobial infections. Twenty randomly selected CTX-M-15-carrying *E. coli *isolates were positive for the 1.8 kb IS*Ecp*1 element using designed primers. The nucleotide sequences of several CTX-M 15 alleles were published at GenBank under the accession numbers EU118591 to EU118602.

### Antibiotic resistance profiles

All isolates were sensitive to imipenem/cilastin and meropenem. Additionally, we tested for tigecycline and obtained MICs ranging from 0.5 μg/ml to 1 μg/ml with a median of 0.75 μg/ml indicating that all isolates were sensitive to tigecycline. All isolates carrying the CTX-M-15 allele were resistant to cefepime (MIC ≥ 8 μg/ml). The majority of isolates carrying CTX-M-15 were resistant to gentamicin, trimethoprim/sulfamethoxazole (SXT), tetracycline and ciprofloxacin when compared to other alleles (p = 0.006, chi-square test). There was a significant association (p = 0.00067, Fisher exact test) between resistance to cefepime and the presence of the CTX-M-15 allele, when compared to other CTX-M alleles data not shown.

### Conjugation

The 18 randomly selected CTX-M-15-carrying *E. coli *isolates were all capable of transferring plasmids by conjugation with transfer frequencies ranging from 10^-4 ^– 10^-9 ^per donor cell. Genes for tetracycline and SXT resistance were co-transferable in 61% and gentamicin in 33% of isolates. No transferable ciprofloxacin resistance was observed (Table 1).

**Table 1:** Characteristic of 18 *Escherichia coli *selected as donors

Isolate	Phylogenetic Group	PFGE type	ESBL type	Resistance to antibiotics other than Beta-lactam	Conjugation frequency	Incompatibility group
12	B1	X13	CTX-M-15	GM,*SXT,*TET,CIP	10^-9^	FIA,FIB
19	A	X13	CTX-M-15	GM,*SXT,TET,CIP	10^-9^	FIA,FIB
44	A	X5	CTX-M-15,TEM-1	GM,CIP	10^-9^	FIA,FIB
48	D	X13	CTX-M-15	*GM,CIP,*SXT,*TET	10^-7^	FIA,FIB
58	B2	X5	CTX-M-15	GM,CIP,SXT	10^-4^	FIA,FIB
66	A	X9	CTX-M-15,TEM-1	*GM,CIP,*TET,*SXT	10^-7^	FIA,FIB
67	A	X9	CTX-M-15,TEM-1	GM,CIP,*TET,*SXT	10^-7^	FIA,FIB
70	B2	X5	CTX-M-15,TEM-1	GM,*SXT,TET,CIP	10^-9^	FIA,FIB
81	D	X6	CTX-M-28	CIP, SXT,*TET	10^-9^	FIB
90	A	X12	CTX-M-3	*GM,CIP,SXT,*TET	10^-9^	FIA,FIB
92	B2	X5	CTX-M-15	GM, TET, SXT	10^-9^	FIA,FIB
95	A	X9	CTX-M-1	SXT	10^-8^	FIA,FIB
103	B2	X12	CTX-M-15,TEM-1	*GM, CIP,*TET, SXT	10^-7^	FIA, FIB
79	B1	X5	CTX-M-15,TEM-1	*GM,CIP,*TET,*SXT	10^-7^	FIA
54	A	X9	CTX-M-15, TEM-1	GM,CIP, *TET, *SXT	10^-6^	FIA
102	B2	X1	CTX-M-15, TEM-1	GM,CIP,*TET, *SXT	10^-7^	FIA
110	D	X4	CTX-M-15	*GM,CIP,*SXT,*TET	10^-9^	FIA,FIB
112	B2	X4	CTX-M-15	GM,CIP,*SXT,TET	10^-9^	FIA,FIB

*Transferable resistance, GM: Gentamicin, TET: Tetracycline, CIP: Ciprofloxacin, SXT: Sulphamethoxazole/Trimethoprim. The rate of transferable antibiotic resistance for GM, SXT, TET, GM-SXT-TET, SXT-TET and GM-TET was 33%, 61%, 61%, 27%, 44% and 11% respectively.

### Characterization of isolates using PFGE and phylogenetic grouping

**Figure 1:**
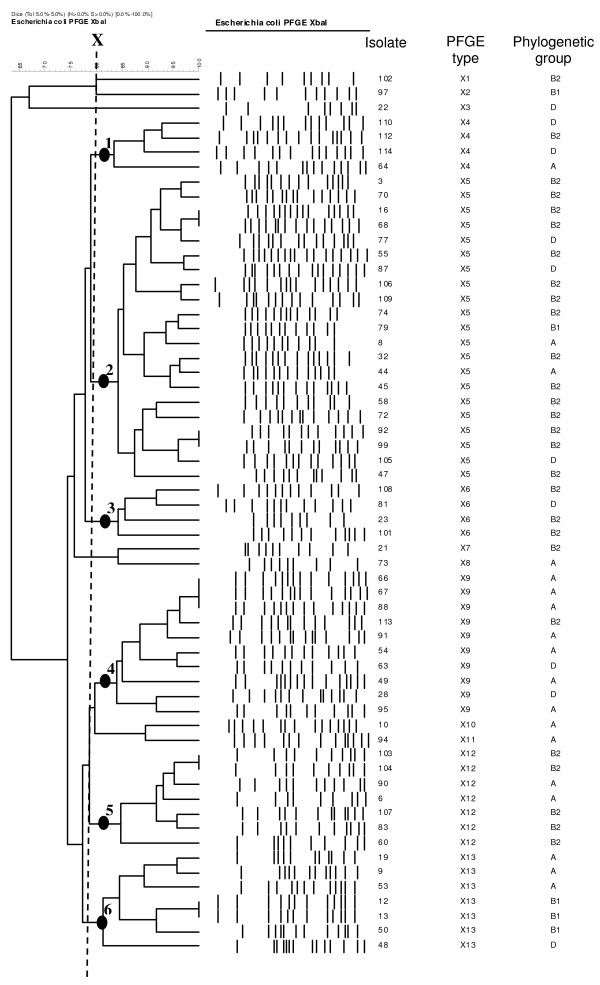
**PFGE dendogram of ESBL-producing *E. coli***. Heterogeneity of the 63 *E. coli *ESBL producers are seen on the dendograms. The diagram also shows the isolate number, PFGE types as well as the corresponding phylogenetic group. The dashed line X indicates SAB of 0.8 revealing six clusters 1–6; PFGE types are labelled X1–X13.

All of the 63 *E. coli *isolates were subjected to PFGE analysis. Examination of the PFGE patterns revealed 13 PFGE types (Figure 1). Using a similarity level SAB of 0.80 (line X in Figure 1), the 63 strains could be assigned to 6 clusters (clusters 1–6 in Figure 1) indicating the overall close relationship of the isolates. PCR-based phylogenetic analysis revealed that 28 (44.4%) of all isolates belonged to the B2 phylogenetic group, 20 were classified as group A (31.7%), 10 (15.8%) as D and 5 (7.9%) as B1. CTX-M-15 was found in 61% of group B1 isolates, in 50% of group D isolates, and in 60% of group A and B2, respectively. Thirteen (65%) of the group A isolates were obtained from urine samples. Phylogenetic group B2 formed the majority of the PFGE clusters 2 (15/21), 3 (3/4) and 5 (5/7) (Figure 1).

### Plasmid analysis and replicon typing

**Figure 2:**
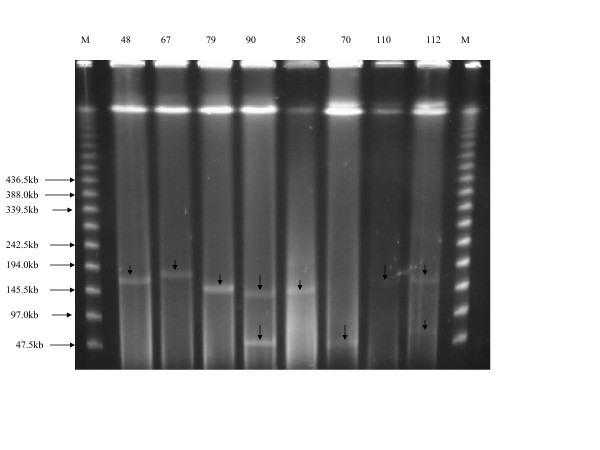
**Agarose gel showing S1 nuclease PFGE-based sizing of large plasmids for 8 isolates**. M (Lambda Marker) indicates the molecular weight marker of concatenated multimers of the bacteriophage lambda genome. Plasmid preparations from isolate number 48, 67, 79, 90, 58, 70, 110 and 112 reveal plasmids with sizes ranging from 45.5 kb to 194 kb which are indicated with arrows.

Plasmids of variable sizes were detected, and the majority of transconjugants had plasmids ranging from 145.5 kb to 194 kb (Figure 2). A 145.5 kb plasmid was detected in 65% of the isolates tested and when present it was always found to hybridize with CTX-M-15, FIA as well as FIB probes, '[see Additional file 2]'. In one isolate the CTX-M-gene was located on a 242.5 kb IncF1 plasmid. All clinical isolates and transconjugants were found to harbour IncFI group plasmids. The majority of isolates (71.4%) were positive for both FIA and FIB. FIA and FIB alone were found in 10 (15.8%) and 8 (12.7%) isolates respectively. Plasmids harbouring Inc FII, IncN and IncI1 replicon types were not detected.

## Discussion

This study provides molecular-epidemiological data on ESBL-carrying *Escherichia coli *in the clinical setting of a University Hospital in Germany. The study demonstrates that CTX-M ESBLs are the most common ESBL types among *E. coli *ESBL producers in our setting. The predominance of CTX-M-15 indicates that this allele might now be as common in Germany as in other European countries (such as UK, Poland, Greece, France, etc.) [3-5,12].

CTX-M-15 ESBL producing isolates displayed a significantly (p = 0.0064) higher level of resistance to ciprofloxacin, gentamicin, tetracycline and trimethoprim/sulfamethoxazole (SXT) as compared to other ESBL alleles. Other studies have also reported co-resistance to tetracycline, aminoglycosides, fluoroquinolones in ESBL-producing organisms [9,12,18,19]. In this study gentamicin, tetracycline and SXT resistance were transferable by conjugation in more than 30% of isolates tested. Isolates harbouring CTX-M-15 were resistant to cefepime with most of them exhibiting a MIC of greater than 32 μg/ml which is significantly higher than that reported for other CTX-alleles (p = 0.00067). It has been demonstrated that CTX-M ESBLs hydrolyze cefepime with higher efficiency compared to other ESBL types [13]; this finding was confirmed in our study. Our data furthermore suggests that among the CTX-M-alleles, CTX-M-15 confers a higher ability to hydrolyze cefepime as compared to other CTX-M-types. In addition to carbapenems, all our ESBL isolates habouring CTX-M types were found to be sensitive to tigecycline with a MIC ≤ 2 μg/ml. This suggests that tigecycline could be a therapeutic alternative to carbapenems in cases of systemic infection due to ESBL producing organisms [11,20].

All phylogenetic groups of *E. coli *were observed in our study with group B2 representing the majority of our isolates [21]. Twenty isolates in our study were classified as phylogenetic group A. Among these 13 (65%) were from urine samples and 60% of them carried the CTX-M-15 allele. Bacteria from the phylogenetic group A are considered as commensals, but they have occasionally been associated with serious nosocomial infections. In this study, one group A CTX-M-15 positive isolate was recovered from a blood culture. In contrast to data reported in other studies, we cannot detect a significant bias in the distribution of CTX-M-15 among the different phylogenetic groups [22].

Analyses of PFGE banding patterns indicate a total of 55 genotypes among the isolates collected (Figure 1), suggesting that the spread of ESBLs is most probably linked to mobile genetic elements. Using a similarity coefficient (SAB) of 0.80, we were able to establish 6 different clusters (1–6) in our isolates. Cluster 2 was the largest cluster and was mainly formed by the B2 phylogenetic group with 67% of the isolates harbouring the CTX-M-15 allele. All isolates were typed for plasmid incompatibility groups and could be classified as FIA- and FIB-replicon types. Unlike most previously published studies [4,5,8], where an association of FII together with FIA and/or FIB was observed, no FII-replicon type could be detected in our isolates. To date there is only a single report from Turkey in which CTX-M-15 of an *E. coli *isolate was associated with an IncFI plasmid [8]. Plasmid analysis revealed that the majority of our transconjugants harboured large plasmids ranging from 145.5 to 194 kb. In 65% of the tested ESBL isolates, a common IncFI plasmid of about 145.5 kb could be demonstrated. One isolate carried a 242.5 kb IncFI plasmid harbouring CTX-M-15, which is the largest IncFI plasmid described in *Escherichia coli *to be associated with CTX-M-15. Most previous studies have found plasmids ranging from 7 to 200 kb in association with CTX-M-15 [3-5]. These findings suggest that in our setting, the CTX-M-15 allele is carried on large conjugative plasmids which are well-adapted and constantly exchanged by lateral gene transfer among the *E. coli *isolates. The detection of other Enterobacteriaceae with the same ESBL type, as demonstrated in the above-mentioned cases of polymicrobial infections, furthermore suggest a species-overlapping transfer of CTX-M-15 (data not shown). Indeed all *E. coli *tested were able to transfer antibiotic resistance by conjugation with a frequency ranging from 10^-4 ^to 10^-9 ^per donor cell, indicating in several cases an at least 100-fold higher frequency compared to previous reports from the UK [18]. In addition CTX-M ESBL genes are often associated with an IS*Ecp1 *element which facilitates its transfer. In this study the IS*Ecpl *element was found in all isolates tested.

## Conclusion

This study demonstrates the predominant presence of CTX-M-15 ESBL producing *E. coli*, commonly associated with a 145.5 kb IncFI plasmid, in our setting. This is the first report of a CTX-M-15 occurrence in Germany associated with large conjugative plasmids with exclusive IncFI replicon-type. This study furthermore illustrates that the prevalence of CTX-M-15 is not due to spread of a single clonal type but is associated with the spread of related *E. coli *isolates. Dissemination of the ESBL phenotype is linked to the lateral transfer of highly adapted IncF1 conjugative plasmid. Based on these findings, larger multi-center studies to determine the molecular epidemiology of *E. coli *ESBL isolates, the distribution of CTX-M ESBL as well as the presence of conjugative plasmids among Enterobacteriaceae in hospital populations are warranted.

## Competing interests

The authors declare that they have no competing interests.

## Authors' contributions

SEM, ED, CI, and TC designed the study, SEM and CI performed the experiments, SEM, CI, ED, HH and TC analyzed the data, TH analyzed sequences, SEM, CI and TC wrote the manuscript which was corrected and approved by all the other coauthors.

## Pre-publication history

The pre-publication history for this paper can be accessed here:

http://www.biomedcentral.com/1471-2334/9/97/prepub

## Supplementary Material

Additional file 1 **Table**: ESBL producing *E. coli*, ESBL allele, Plasmid incompatibility groups and antibiotic-susceptibility results. Click here for file

Additional file 2 **Figure, A: Agarose gel showing S1 nuclease PFGE-based sizing of large plasmids for 8 isolates**. M (Lambda Marker) indicates the molecular weight marker of concatenated multimers of the bacteriophage lambda genome. A total of eight *E. coli *CC118 transconjugants from conjugation experiments with different isolates are depicted. Plasmids sizes range from 45.5 kb to 194 kb **B: **The corresponding gel following DNA transfer to a polyvinyl-derived membrane and subsequent hybridization with the digioxygenin (DIG)-labelled CTX-M-15 probe. Click here for file
