# Supplementary material for: Conjugative IncFI plasmids carrying CTX-M-15 among Escherichia coli ESBL producing isolates at a University hospital in Germany
Source: BMC Infect Dis. 2009 Jun 17;9:97. doi: 10.1186/1471-2334-9-97 (PMC2708165; doi:10.1186/1471-2334-9-97)
Supplement: Additional file 1 — Table: ESBL producing E. coli, ESBL allele, Plasmid incompatibility groups and antibiotic-susceptibility results. [file 1471-2334-9-97-S1.doc]

TABLE 2: ESBL producing *E. coli*, ESBL allele, Plasmid incompatibility groups and antibiotic-susceptibility results

| Isolate | Ward | Specimen | ESBL type | MIC µg/ml | | Resistance to antibiotics other than Beta-lactams | Incomp-atibility group | Phylo. group | PFGE  type |
| --- | --- | --- | --- | --- | --- | --- | --- | --- | --- |
|  |  |  |  | FEP | TGC |  |  |  |  |
| 3 | Urology | Blood | Tem-144 | >256 | - | GM, CIP, SXT, TET | FIA, FIB | B2 | X5 |
| 6 | Urology | Urine | CTX-M-15 | 256 | 0.75 | GM, CIP, SXT, | FIA, FIB | A | X12 |
| 8 | Surgical | Urine | CTX-M-15,Tem1b | 16 | 1.5 | GM, CIP, SXT, TET | FIA, FIB | A | X5 |
| 9 | Surgical | W. swab | Tem-144 | 256 | - | GM, SXT, TET | FIA, FIB | A | X13 |
| 10 | Surgical | W. swab | CTX-M-15 | >256 | 1.5 | GM, SXT, TET, CIP | FIA, FIB | A | X10 |
| 12 | ICU | Tra. swab | CTX-M-15 | 128 | 0.75 | GM, SXT, TET, CIP | FIA, FIB | B1 | X13 |
| 13 | ICU | Tra. swab | CTX-M-15 | 64 | 0.75 | GM, SXT, TET, CIP | FIA, FIB | B1 | X13 |
| 16 | Int. Medicine | Urine | CTX-M-15,Tem-1 | 256 | 1.5 | GM, SXT, TET, CIP | FIA, FIB | B2 | X5 |
| 19 | Int. Medicine | W. swab | CTX-M-15 | 32 | 0.75 | GM, SXT, TET, CIP | FIA, FIB | A | X13 |
| 21 | OB/GY | CX. swab | Tem144 | 48 | - | CIP, TET | FIA | B2 | X7 |
| 22 | Int. Medicine | Phar. swab | CTX-M-3 | 32 | 0.75 | CIP, SXT, TET | FIA, FIB | D | X3 |
| 23 | Paediatric | Blood | Tem-150 | 8 | - | GM, CIP, TET, SXT | FIB | B2 | X6 |
| 26 | Surgical | Urine | CTX-M-15,Tem-1 | 48 | 2 | GM, CIP, TET, SXT | FIA | B2 | ND |
| 28 | OB/GY | Vag. swab | Tem-144 | 16 | - | GM, CIP, TET, SXT | FIA | D | X9 |
| 38 | Int. Medicine | Urine | CTX-M-1, Tem-1 | ND | 0.25 | SXT, TET | FIA | B2 | ND |
| 44 | Urology | Urine | CTX-M-15,Tem-1 | 256 | 0.75 | GM, CIP | FIA, FIB | A | X5 |
| 45 | Urology | Urine | Tem-144 | 128 | - | CIP, SXT | FIA, FIB | B2 | X5 |
| 47 | Int. Medicine | Urine | CTX-M-1 | 64 | - | NONE | FIA, FIB | B2 | X5 |
| 48 | ICU | Sputum | CTX-M-15 | 64 | 0.5 | GM, CIP, SXT, TET | FIA, FIB | D | X13 |

TABLE 1 continued

| *ISOLATE* | Ward | Specimen | ESBL type | MIC µg/ml  FEP TGC | | Resistance to antibiotics other than Beta-lactams | Incomp-atibility group | Phylo.group | PFGE group |
| --- | --- | --- | --- | --- | --- | --- | --- | --- | --- |
| 49 | Int. Medicine | Phar. swab | CTX-M-1, Tem-1 | 256 | 1.0 | TET, SXT | FIB | A | X9 |
| 50 | Paediatric | Eye swab | CTX-M-1, Tem-1 | 2 | 1.0 | TET, SXT | FIA, FIB | B1 | X13 |
| 53 | OB/GY | CX. swab | CTX-M-28,Tem-1 | 32 | 1.5 | GM, CIP, TET, SXT | FIA, FIB | A | X13 |
| 54 | Orthopaedic | Urine | CTX-M-15,Tem-1 | 32 | 0.5 | GM, CIP, TET, SXT | FIA, FIB | A | X9 |
| 55 | Int. Medicine | Blood | CTX-M-15 | 256 | 0.5 | GM, CIP, SXT, TET | FIA, FIB | B2 | X5 |
| 58  60 | Urology  Int. Medicine | Urine  Sputum | CTX-M-15  Tem-143 | >256  12 | 0.5  0.25 | GM, CIP, TET  GM, CIP, SXT | FIA, FIB  FIA, FIB | B2  B2 | X5  X12 |
| 63 | ICU | Urine | CTX-M-15,Tem-1 | 256 | 0.73 | GM, CIP, TET, SXT | FIA, FIB | D | X9 |
| 64 | Int. Medicine | Urine | CTX-M-15 | 48 | 0.75 | GM, CIP, TET, SXT | FIA, FIB | A | X4 |
| 66 | Int. Medicine | Urine | CTX-M-15,Tem-1 | 256 | 0.5 | GM, CIP,TET**,**SXT | FIA, FIB | A | X9 |
| 67 | ICU | Urine | CTX-M-15,Tem-1 | 64 | 0.5 | GM, CIP, TET, SXT | FIA, FIB | A | X9 |
| 68 | Urology | Urine | CTX-M-15,Tem-1 | 64 | 0.25 | GM, CIP, TET, SXT | FIA, FIB | B2 | X5 |
| 70 | Urology | Urine | CTX-M-15,Tem-1 | >256 | 2 | GM, CIP, TET, SXT | FIA, FIB | B2 | X5 |
| 72 | Paediatric | Urine | CTX-M-1,Tem-1 | 48 | 0.25 | SXT, TET | FIA | A | X5 |
| 73 | Int. Medicine | Urine | CTX-M-1,Tem-1 | 2 | 0.25 | GM, CIP, TET, SXT | FIB | A | X8 |
| 74 | Surgical | W. swab | CTX-M-15,Tem-1 | >256 | 0.5 | GM, CIP, TET, | FIA | B2 | X5 |
| 77 | Paediatric | Urine | Tem-143 | 2 | - | GM, CIP, TET, SXT | FIB | D | X5 |
| 81 | Int. Medicine | Urine | CTX-M-28,Tem-1 | 0.064 | 0.5 | CIP, TET, SXT | FIB | D | X6 |
| 83 | Int. Medicine | Urine | CTX-M-15,Tem-1 | 128 | 0.38 | GM, CIP, TET | FIA, FIB | B2 | X12 |
| 87 | Int. Medicine | Eye swab | CTX-M-15,Tem-1 | 64 | 0.5 | GM, SXT, TET, CIP | FIA, FIB | D | X5 |
| 88 | Int. Medicine | Urine | CTX-M-15,Tem-1 | 64 | 0.38 | GM, SXT, TET, CIP | FIA, FIB | A | X9 |
| 90 | Int. Medicine | Urine | CTX-M-3 | 256 | 0.75 | GM, CIP, TET, SXT | FIA, FIB | A | X12 |
| 91 | Urology | Urine | Tem-105 | 12 | - | CIP, SXT, TET | FIB | A | X9 |
| 92 | Paediatric | Urine | CTX-M-15 | >256 | 0.75 | GM, TET, SXT | FIA, FIB | B2 | X5 |
| 94 | Int. Medicine | Blood | CTX-M-15 | 24 | 0.5 | GM, CIP, SXT, TET | FIA, FIB | A | X11 |

TABLE 1 continued

| *ISOLATE* | Ward | Specimen | ESBL type | MIC µg/ml  FEP TGC | | Resistance to antibiotics other than Beta-lactams | Incomp-atibility group | Phylo. group | PFGE group |
| --- | --- | --- | --- | --- | --- | --- | --- | --- | --- |
| 95 | Surgical | W. swab | CTX-M-1 | 4 | 0.25 | SXT | FIA, FIB | A | X9 |
| 97 | Int. Medicine | Sputum | Tem-105 | 16 | - | GM, CIP, SXT, TET | FIA, FIB | B1 | X2 |
| 99 | Int. Medicine | Urine | CTX-M-15,Tem-1 | 48 | 0.75 | CIP, TET, SXT | FIA | B2 | X5 |
| 101 | ICU | Urine | CTX-M-15,Tem-1 | 32 | 0.75 | GM, TET, SXT | FIA, FIB | B2 | X6 |
| 102 | Surgical | Sputum | CTX-M-15,Tem-1 | 48 | 1.0 | GM, TET, SXT, CIP | FIA, FIB | B2 | X1 |
| 103 | Int. Medicine | Urine | CTX-M-15,Tem-1 | 64 | 1.0 | GM, CIP, TET, SXT | FIA, FIB | B2 | X12 |
| 104 | Int. Medicine | Urine | CTX-M-15 | 32 | 0.75 | GM, CIP, TET, SXT | FIA, FIB | B2 | X12 |
| 105 | Int. Medicine | Urine | NEG | 32 | - | GM, CIP, TET, SXT | FIA, FIB | D | X5 |
| 106 | Int. Medicine | Urine | CTX-M-15,Tem-1 | 64 | 0.75 | GM, CIP, TET, SXT | FIA | B2 | X5 |
| 107 | Int. Medicine | Swab | Negative |  | - | GM, CIP, TET, SXT | FIB | B2 | X12 |
| 108 | Int. Medicine | CX. swab | Tem-126 | 256 | - | GM, CIP, SXT | FIA, FIB | B2 | X6 |
| 109 | Int. Medicine | Blood | CTX-M-3 | 2 | 0.75 | CIP, TET, SXT | FIA | B2 | X5 |
| 110 | Urology | Urine | CTX-M-15 | 64 | 0.75 | GM, CIP, TET, SXT | FIA, FIB | D | X4 |
| 112 | Int. Medicine | Urine | CTX-M-15 | >256 | 1.0 | GM, CIP, TET, SXT | FIA, FIB | B2 | X4 |
| 113 | Urology | Urine | Tem-126 | 16 | 0.75 | GM, CIP, TET, SXT | FIB | B2 | X9 |
| 114 | Urology | Urine | CTX-M-15,Tem-1 | 256 | 0.5 | GM, CIP, TET, SXT | FIA, FIB | D | X4 |
| 79 | ICU | Blood | CTX-M-15,Tem-1 | 48 | 0.5 | GM, CIP, TET, SXT | FIA | B1 | X5 |
| 27 | Int. Medicine | Urine | CTX-M-3 | - | - | GM, CIP, TET, SXT | FIA, FIB | A | ND |
| 32 | Int. Medicine | Sputum | CTX-M-15 | >256 | 1.0 | GM, CIP, TET, SXT | FIA, FIB | B2 | X5 |

GM: Gentamicin, TET: Tetracycline, CIP: Ciprofloxacin, SXT: Sulphamethoxazole/Trimethoprim, FEP: Cefepime. TGC: Tigecycline (determined only for CTX-M positive isolates), ND/ -: not determined, W.: Wound, CX.: Cervix, Phar.: Pharyngeal, Vag.: Vaginal, Tra.: Tracheal, Int.: Internal, ICU: Intensive care unit, OB/GY: Obstetrics/Gynecology, Phylo. : Phylogenetic
