# Supplementary figures and images for: Conjugative IncFI plasmids carrying CTX-M-15 among Escherichia coli ESBL producing isolates at a University hospital in Germany
Source: BMC Infect Dis. 2009 Jun 17;9:97. doi: 10.1186/1471-2334-9-97 (PMC2708165; doi:10.1186/1471-2334-9-97)

## Slide 1
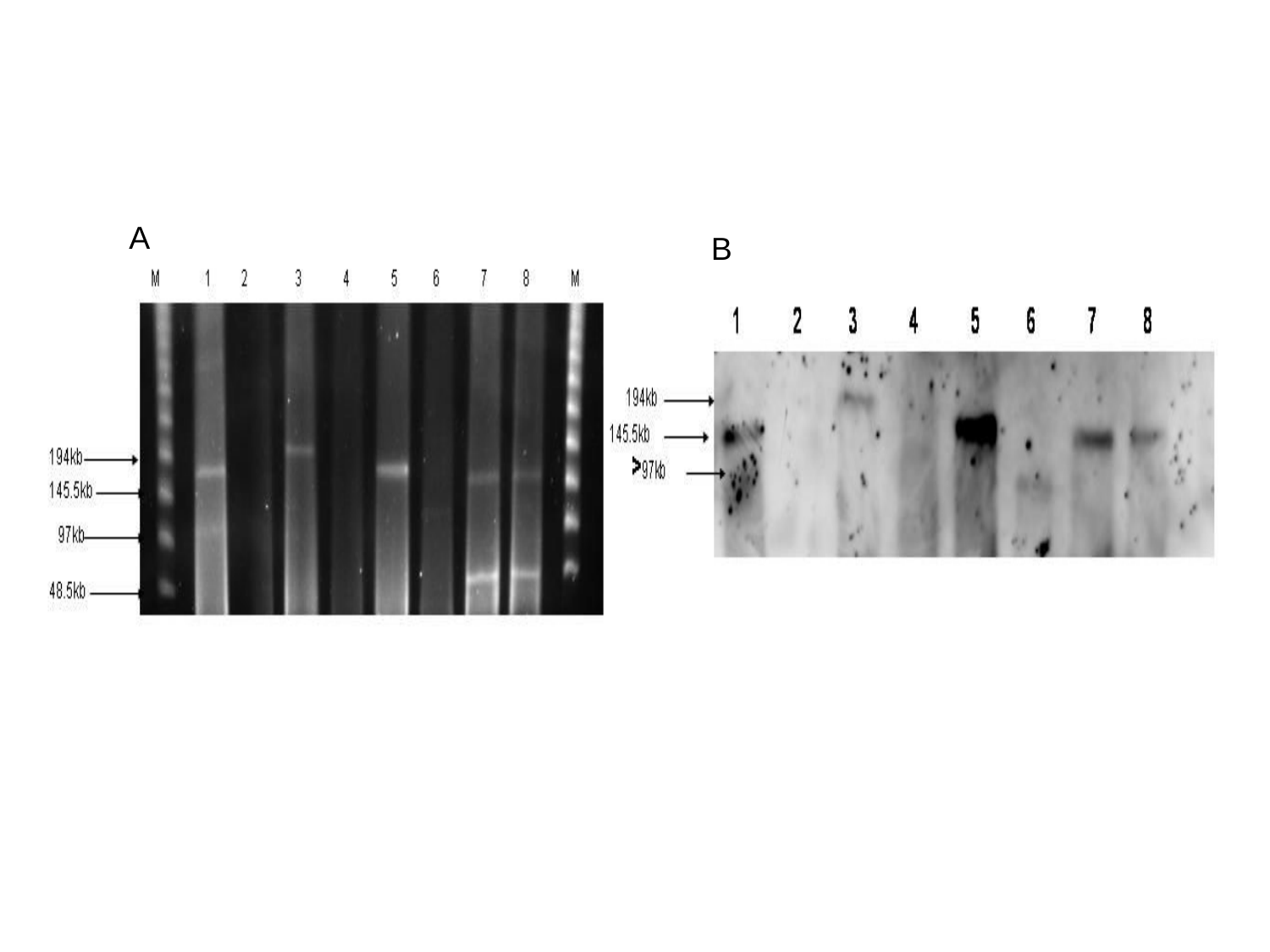

A
B

Supplement: Additional file 2 — Figure, A: Agarose gel showing S1 nuclease PFGE-based sizing of large plasmids for 8 isolates. M (Lambda Marker) indicates the molecular weight marker of concatenated multimers of the bacteriophage lambda genome. A total of eight E. coli CC118 transconjugants from conjugation experiments with different isolates are depicted. Plasmids sizes range from 45.5 kb to 194 kb B: The corresponding gel following DNA transfer to a polyvinyl-derived membrane and subsequent hybridization with the digioxygenin (DIG)-labelled CTX-M-15 probe. [file 1471-2334-9-97-S2.ppt]
